# Iron Status in Sickle Cell Anemia: Deficiency or Overload?

**DOI:** 10.7759/cureus.35310

**Published:** 2023-02-22

**Authors:** Varsha P Patel, Prayag R Pandya, Darshankumar M Raval, Princy D Lukhi, Vaishnavi M Rathod, Shahin Khan, Shashwat Mallik, Amoghavarsha Venugopal, Anek Jena, Kush Patel, Dirgha Patel, Riya Dobariya

**Affiliations:** 1 Internal Medicine, Government Medical College, Baroda, Vadodara, IND; 2 Internal Medicine, GMERS (Gujarat Medical Education and Research Society) Medical College, Gotri, Vadodara, IND; 3 Medicine, Government Medical College, Baroda, Vadodara, IND; 4 Infectious Disease, Mayo Clinic, Jacksonville, USA; 5 Critical Care, Mayo Clinic, Jacksonville, USA

**Keywords:** hemoglobinopathy, target organ damage, iron overload, sickle cell anemia, hematology, internal medicine

## Abstract

Background

Sickle cell anemia (SCA) is a hereditary disease with defective hemoglobin (Hb) synthesis causing severe hemolytic anemia, pain crisis, and target organ damage. In SCA, several factors independently or in combination lead to derangement in iron stores. Some centers incorrectly prescribe iron therapy on the presumption that SCA would be associated with iron deficiency, but it is not always the case. This study attempts to evaluate the iron status in SCA patients and records the target organ damage present.

Methodology

A single-center cross-sectional study of 180 patients with sickle cell disease was carried out at a tertiary-care center in Western India. Patients >12 years of age were included in the study after confirming SCA using high-performance liquid chromatography (HPLC). The iron status of each patient was identified and patients were labeled as iron sufficient based on the following values: Hb (8.1-12 gm%), serum iron (S. iron) level (50-150 μg/dl), serum ferritin (S. ferritin) (50-200 ng/ml), and total iron binding capacity (TIBC) (251-450 µg/dl). The iron status of patients with different target organ damage was also analyzed.

Results

Demographic data revealed that 21-30 years was the most common age group affected by SCA along with a male preponderance. The most common presenting complaint was joint pain (68.9%), the most common sign was pallor (64.4%), most patients had a history of pain crisis (95.6%), and half of the patients had organomegaly (51.1%). Most of the patients had no complications, however, for those who did, hepatopathy (28.9%) was the most common.

Conclusion

While the majority of patients were iron sufficient, a considerable number had either iron deficiency or iron overload states, which emphasizes the necessity of investigating the iron status before deciding the course of treatment in SCA patients. Although the majority were unaffected, screening for end-organ damage should be carried out in all SCA patients.

## Introduction

Sickle cell anemia (SCA) is an autosomal recessively inherited disease due to a genetic defect in the beta-globin chain of hemoglobin (Hb). It is estimated that 7% of the world's population carries an abnormal Hb gene while approximately 300,000 to 500,000 are born each year with significant Hb disorders. They mainly consist of two groups: thalassemia and sickle cell syndromes. Among those hemoglobinopathies, sickle cell syndromes are more prevalent consisting of 70% of all hemoglobinopathies worldwide [1]. The prevalence of SCA in Western India is around 6.5%, and the majority (up to 30-40%) is reported from tribal areas of Gujarat [2].

The clinical manifestations of SCA result from increased blood viscosity and vascular obstruction by deformed sickled red blood cells (RBC). Reduced flexibility of RBC leads to stroke, splenic sequestration crisis, aplastic crisis, infections, and bone damage. Patients with SCA have higher body concentrations of iron than non-sickle cell anemia. This has been primarily attributed to frequent transfusions, increased gastrointestinal absorption of iron, and chronic hemolysis [3]. Iron overload produces toxicity and cell death through free radical formation and lipid peroxidation. On the other hand, overt iron deficiency with low serum ferritin (S. ferritin) can occur in SCA despite marked hemosiderosis [4]. The excess iron deposits are unavailable for erythropoiesis and, hence, may not be reflected in S. ferritin levels. However, iron deficiency anemia can co-exist in patients with SCA, as they are not immune to environmental factors that precipitate iron deficiency anemia. These factors, especially in the tropics, include poor nutrition, parasitic infestations, and various bacterial infections, which can impair iron metabolism [5]. Also, excessive iron loss in the urine, poor iron absorption and metabolism due to multiple mucosal/submucosal infarctions, and progressive damage of several organs make patients very susceptible to iron deficiency anemia [5,6]. Iron deficiency or iron overload, which complicates SCA, is likely to worsen the clinical condition [7]. Therefore, iron hemostasis requires strict regulation to avoid complications [8].

In patients with sickle cell disease, conventional laboratory tests for iron deficiency, such as S. ferritin, total iron binding capacity (TIBC), transferrin saturation, and mean corpuscular volume (MCV), may be abnormal. The present study aimed to assess the baseline iron status using serum iron (S. iron), S. ferritin, and serum TIBC in ambulatory patients with sickle cell disease. We also evaluated the presence of target organ damage, such as liver, kidney, heart, and pancreas, in SCA patients with varied S. ferritin levels.

## Materials and methods

An observational cross-sectional study was conducted in the department of general medicine over a duration of one year, after receiving approval from the institutional ethics committee (IECBHR/54-2020). A total of 180 patients were included who fulfilled the inclusion criteria. Both male and female patients >12 years of age, with a known case of sickle cell disease or diagnosed with sickle cell disease with high-performance liquid chromatography (HPLC) results showing homozygous HbSS or HbSF were included. Written informed consent was obtained from the patient or legal guardian prior to recruitment. All patients who were less than 12 years of age, with sickle cell trait, pregnant females, those on iron chelation therapy, or with a history of blood transfusion in the past three months were excluded from the study. SCA was diagnosed by the value of the proportion of HbS. HbS produced should be more than 50% of the total Hb to define SCA [9]. In known cases of SCA, the HPLC test was not repeated before inclusion in the study.

After selection, a detailed medical history was taken for all the patients. Sociodemographic data, age, sex, education, marital status, and place of birth were documented. Any history of blood transfusion and chelation therapy was also recorded. The basic details, including diagnosis, medications, and transfusion history, were obtained by reviewing patients' medical records. The history was followed by a general and systemic examination. Patients were inquired about the history of sickle cell crisis, which involves a vaso-occlusive crisis causing pain in the bones, arms, legs, back, knees, and stomach. Investigations including complete blood count (CBC), RBC indices, iron studies, peripheral blood smear, and tests for target organ damage were done. Patients were labeled as iron sufficient based on the following values: S. iron (50-150 μg/dl), S. ferritin (50-200ng/ml), and TIBC (251-450µg/dl) [10]. Patients were classified into mild (10-12 gm%), moderate (8-10 gm%), and severe anemia (<8 gm%).

## Results

The average age of patients in the study was 25.4 years. The majority of patients (n=72, 40%) in the study were between 21 and 30 years of age. The number of males (n=116, 64.4%) was twice the number of females (n=64, 35.6%). Out of 140 patients, 124 (68.9%) had a presenting complaint of joint pain out of which 33.33% of patients (n=60) had Hb less than 8.1 gm%. The second most common presenting feature was yellowish discoloration of the urine and/or sclera (n=44, 24.4%) followed by shortness of breath (n=12, 6.7%) and abdominal pain (n=8, 4.4%) (Table 1). This indicates that vaso-occlusive crisis was the most commonly encountered sickle cell crisis in our study population, followed by hemolytic crisis and acute chest syndrome.

**Table 1:** Presenting complaints of patients

Presenting Complaints	Number of patients
Joint Pain	124
Yellowish Discoloration of Urine and/or Sclera	44
Breathlessness	12
Abdominal Pain	8
Palpitation	4
Pedal Edema	4
Polyuria	4
Polydipsia	4
Polyphagia	4
Vomiting	4
Decreased Urine Output	4

The most frequently observed sign was pallor (n=116, 64.4%), followed by icterus (n=68, 37.8%). Forty-eight point two eight percent (48.28%; 56) patients with pallor and 29.41% (20) patients with icterus had Hb less than 8.1 gm%. This finding suggests that pallor was a more commonly observed sign than icterus in severely anemic patients. Clubbing and edema were present in 13.3% (n=24) and 4.4% (n=8) of patients, respectively. Ninety-five point six (95.6%; n=172) of the patients had a history of pain crisis, out of which 51.13% (n=88) were moderately anemic. On average, there were 3.6 episodes of joint pain in each patient. A history of blood transfusion was present in 120 (66.7%) patients with a mean of 2.4 blood transfusions per patient. A past history of jaundice was reported in 72 (40%) patients, out of which 50% (n=36) had S. iron >150 μg/dL. On average, nearly one episode of jaundice was noted in the past in each patient. A history of acute chest syndrome episodes and avascular necrosis episodes was present in 10 (22.2%) and eight (17.8%) patients respectively (Table 2).

**Table 2:** Past history of patients

Past History	Number of Patients
Pain Crisis	172 (95.6%)
Blood transfusion	120 (66.7%)
Jaundice	72 (40.0%)
Acute Chest syndrome	40 (22.2%)
Avascular necrosis	32 (17.8%)

On per abdominal examination of patients, organomegaly (liver, spleen, or both) was reported in more than half (n=92, 51.1%) of the patients. Hepatomegaly and splenomegaly were reported in 24.4% (n=44) and 20% (n=36) of patients respectively while hepatosplenomegaly was found in 6.7% (n=12) patients. Sixty percent (60%) of the patients had mild to moderate degrees of anemia. Of the total patients with a history of pain crisis (172), 58.13% had mild to moderate anemia and 2.3% had normal Hb. Among the patients with a history of blood transfusion, 60% had mild to moderate anemia (Table 3).

**Table 3:** Descriptive statistics of Hb in patients Hb = hemoglobin

Hb level (gm%)	Number of patients	Number of patients with pain crisis	Number of patients with a history of blood transfusion
≤ 8	68 (37.8%)	68 (37.8%)	44 (36.66%)
8.1-12	108 (60.0%)	100 (58.13%)	72 (60.0%)
>12	4 (2.2%)	4 (2.3%)	4 (3.33%)
Total	180	172	120

The iron studies showed that S. iron was deficient in 56 (31.1%), normal in 84 (46.7%), and elevated in 40 (22.2%) patients. Among patients with raised S. iron (n=40), 36 (90%) had jaundice. This showed that almost half of the patients had sufficient iron stores; whereas, among patients with high iron stores, jaundice was a prevalent feature. Thirty-one point one percent (31.1%) of patients (n=56) had reduced, 40% (n=72) had normal, and 28.8% (n=52) had raised S. ferritin levels. TIBC was seen to be reduced in 28.9% (n=52), normal in 37.38% (n=68), and raised in 33.3% (n=60) of patients.

**Figure 1:**
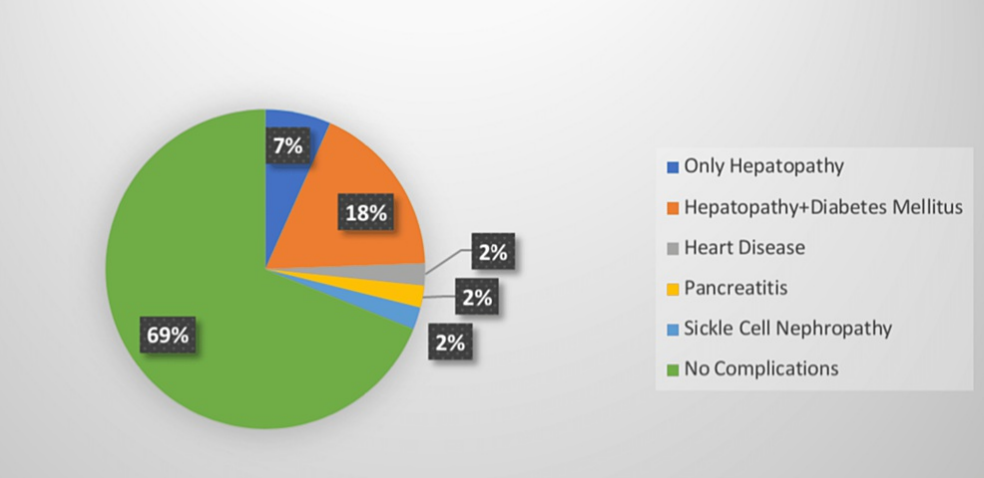
Complications due to iron overload

**Figure 2:**
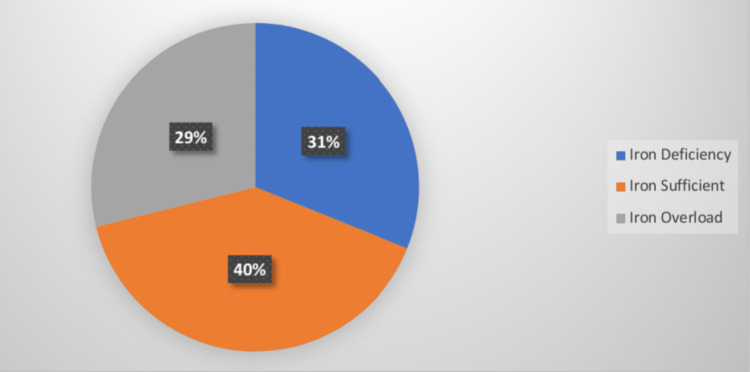
Iron storage status in patients

Hepatopathy was the most common complication and was seen in 44 patients (Figure 1). Among 44 patients, 32 had high, eight had low, and four had normal S. ferritin levels. Out of 124 patients who had no complications, 68 had normal, 44 had low, and 12 had high S. ferritin levels. Patients with heart disease (n=4) and sickle cell nephropathy (n=4) had high S. ferritin while low S. ferritin was seen in patients with pancreatitis (n=4). Most patients (40%) were iron sufficient. Iron deficiency was seen in 56 (31.1%), and iron overload was seen in 52 (28.9%) patients with sickle cell disease (Figure 2).

## Discussion

In the present study, sickle cell disease was more common among males (64.4%) than females (35.6%), similar to a cross-sectional study conducted by Brahme et al. in Gujarat, where 56.1% of patients were male and 43.9% patients were female [11]. This suggests a slightly higher prevalence of sickle cell disease in males despite females being at higher risk of developing anemia due to blood loss during menstruation and childbirth apart from nutritional deficiency. In contrast to the present study, where most patients were between 21 and 30 years of age (40%), the majority (48.8%) of the patients belonged to the 12-20 years age group in the study conducted by Brahme et al. [11].

In our study, the majority of the patients (68.9%) were having joint pain at the time of presentation, followed by yellowish discoloration of urine and/or sclera, which was observed in 44 (24.4%) patients. Breathlessness and abdominal pain were observed in 12 (6.7%) patients and eight (4.4%) patients, respectively. Similar findings have been noted in a study conducted by Patel et al. among sickle cell disease patients in Gujarat, where the most common presenting complaint was musculoskeletal pain (83.61%), followed by yellow discoloration of the sclera and/or urine (67.21%), fever (65.57%), breathlessness (54.1%), abdominal pain (48.34%), and chest pain (31.67%) [12]. In the present study, past history of pain crisis was seen in 95.6% of patients while a history of blood transfusion was present in 66.7% of patients. History of jaundice, acute chest syndrome episode, and avascular necrosis episode was present in 40%, 22.2%, and 17.8% of patients, respectively. However, in a study conducted by Patel et al., a past history of pain crises was seen in 73.33% of patients while a past history of jaundice and blood transfusion was seen in 63.33% and 31.67% of patients [12]. Acute chest syndrome and avascular necrosis were seen in 30% and 6.56% of patients, respectively, in a study conducted by Patel et al. [12]. In the present study, organomegaly was reported in 92 (51.1%) patients while 88 (48.9%) patients had no organomegaly. Hepatomegaly and splenomegaly were reported in 24.4% and 20% of patients, respectively. Hepatosplenomegaly was reported in 6.7% of patients. In a study conducted by Yadav et al., organomegaly was reported in a higher proportion (70%) of patients than in the present study (51.1%) [13].

Although a large number of patients (37.8%) had Hb less than 8.1 gm%, the majority of the patients (60%) had Hb between 8.1 to 12 gm% in our study. Therefore, in the present study, more than half of patients (62.2%) had a Hb of more than 8 gm% out of which only four (2.2%) patients had a Hb of more than 12 gm%. However, in a study conducted by Brahme et al., only 21.95% of patients had Hb levels of more than 9 gm% [11].

In the present study, S. ferritin was less than 50 ng/ml in 31.1% of patients, between 50 and 200 ng/ml in 40% of patients, while in 28.8% of patients, S. ferritin was >200 µg/dl. A study conducted by Mohanty P et al. showed an S. ferritin value of less than 20 ng/ml in 9.6% of patients, between 20 and 300 ng/ml in 46.2% of patients, and a value of more than 300 ng/ml in 44.1% of patients [14]. In our study, 46.7% of patients had adequate iron stores, followed by 31.1% of patients with low iron and 22.2% of patients with high iron levels.

In the present study, 31.1% of patients had complications while in 68.9% of patients, no complications were found. Hepatopathy was a more common complication seen in 24.4% of sickle cell disease patients in our study. A study by Patel et al. reported 10% of patients with hepatopathy [12]. Hepatomegaly in a later stage can lead to cirrhosis as evidenced by some autopsy series. Some studies reported a 16% to 29% prevalence of cirrhosis in SCA patients [15-17]. The present study reported diabetes mellitus with hepatopathy in 17.8% of patients, and heart disease and pancreatitis in 4.4% of patients.

Most of the patients (40%) were iron sufficient in our study. The iron deficiency (n=56, 31.1%) and iron overload (n=52, 28.9%) states were almost equally distributed in the study population with a slightly higher prevalence of iron deficiency. A study by Mohanty P et al. reported the majority of patients (80.8%) were iron sufficient, and only a few patients with iron overload and iron deficiency had 9.6% of patients in each group [14], which was lower compared to our study. In contrast to the above studies, a study by Mohanty D et al. reported iron deficiency anemia in 67.7% of sickle cell disease patients [18]. But this could either be a consequence of SCA or a coincidental association due to other factors causing iron deficiency.

This is a single-center study involving a limited population and, hence, generalization of the results to a wider population requires larger multicentric studies. The patients in our study had a history of blood transfusions more than three months back, which could affect the study results. S. ferritin is not the best investigation to determine the iron status and the more definitive method is a bone marrow examination, which was not done in our study.

## Conclusions

Of 180 sickle cell disease patients, the majority of the patients were between 21 and 30 years of age. Although females are more prone to anemia due to several risk factors, such as childbirth, menstrual blood loss, and poor nutrition, in our study, a male preponderance was noted. Joint pain was the most common presenting complaint, of which one-third had severe anemia; whereas pallor was the most frequent sign, of which almost half of the patients had mild-moderate anemia. Almost all the patients had a history of pain crisis, of which half of the patients had mild-moderate anemia. Half of the patients with a history of jaundice had S. iron levels on the higher side (>150 µg/dl). Half of the study population had organomegaly. Most of the patients did not have any complications, however, among those who did, hepatopathy (with diabetes) was the most common. These indicate the importance of screening for end-organ damage in patients with sickle cell disease at regular intervals, starting at a young age. Most patients had sufficient iron stores followed by an almost equal number with iron deficiency and iron overload states. This indicates that most patients had sufficient iron stores in spite of having sickle cell disease. Therefore, iron studies should be done on every patient with sickle cell anemia before deciding the course of treatment with iron therapy or chelation therapy, as both iron deficiency and iron overload states are dangerous for these patients. Thus, our study emphasizes the role of iron study and screening for target-organ damage in patients with sickle cell disease.
